# A New Clade of Insect-Specific Flaviviruses from Australian *Anopheles* Mosquitoes Displays Species-Specific Host Restriction

**DOI:** 10.1128/mSphere.00262-17

**Published:** 2017-07-12

**Authors:** Agathe M. G. Colmant, Jody Hobson-Peters, Helle Bielefeldt-Ohmann, Andrew F. van den Hurk, Sonja Hall-Mendelin, Weng Kong Chow, Cheryl A. Johansen, Jelke Fros, Peter Simmonds, Daniel Watterson, Chris Cazier, Kayvan Etebari, Sassan Asgari, Benjamin L. Schulz, Nigel Beebe, Laura J. Vet, Thisun B. H. Piyasena, Hong-Duyen Nguyen, Ross T. Barnard, Roy A. Hall

**Affiliations:** a School of Chemistry and Molecular Biosciences, The University of Queensland, St. Lucia, Queensland, Australia; b Australian Infectious Diseases Research Centre (AIDRC), The University of Queensland, St. Lucia, Queensland, Australia; c Public Health Virology, Forensic and Scientific Services, Department of Health, Coopers Plains, Queensland, Australia; d Australian Army Malaria Institute, Gallipoli Barracks, Enoggera, Queensland, Australia; e School of Pathology and Laboratory Medicine, The University of Western Australia, Nedlands, Western Australia, Australia; f PathWest Laboratory Medicine WA, Nedlands, Western Australia, Australia; g Nuffield Department of Medicine, University of Oxford, Oxford, England, United Kingdom; h Technical Services, Biosciences Division, Faculty of Health, Queensland University of Technology, Gardens Point Campus, Brisbane, Queensland, Australia; i School of the Biological Sciences, The University of Queensland, St. Lucia, Queensland, Australia; j CSIRO, Dutton Park, Queensland, Australia; University of Pittsburgh

**Keywords:** *Anopheles*, insect-specific flavivirus, coevolution, dinucleotide analysis, host restriction, immunohistochemistry, monoclonal antibodies, mosquito midgut, recombinant NS1, siRNA

## Abstract

Flaviviruses are arthropod-borne viruses found worldwide and are responsible for significant human and veterinary diseases, including dengue, Zika, and West Nile fever. Some flaviviruses are insect specific and replicate only in mosquitoes. We report a genetically divergent group of insect-specific flaviviruses from *Anopheles* mosquitoes that do not replicate in arthropod cell lines or heterologous *Anopheles* species, exhibiting unprecedented specialization for their host species. Determination of the complete sequences of the RNA genomes of three of these viruses, Karumba virus (KRBV), Haslams Creek virus, and Mac Peak virus (McPV), that are found in high prevalence in some *Anopheles* mosquito populations and detection of virus-specific proteins, replicative double-stranded RNA, and small interfering RNA responses in the host mosquito species provided strong evidence of a functional replicating virus in the mosquito midgut. Analysis of nucleotide composition in the KRBV and McPV sequences also revealed a pattern consistent with the virus evolving to replicate only in insects. These findings represent a significant advance in our knowledge of mosquito-borne flavivirus ecology, host restriction, and evolution.

**IMPORTANCE** Flaviviruses like dengue, Zika, or West Nile virus infect millions of people each year and are transmitted to humans via infected-mosquito bites. A subset of flaviviruses can only replicate in the mosquito host, and recent studies have shown that some can interfere with pathogenic flaviviruses in mosquitoes and limit the replication and transmission of the latter. The insect-specific flaviviruses (ISFs) reported here form a new *Anopheles* mosquito-associated clade separate from the *Aedes*- and *Culex*-associated ISF clades. The identification of distinct clades for each mosquito genus provides new insights into the evolution and ecology of flaviviruses. One of these viruses was shown to replicate in the midgut of the mosquito host and exhibit the most specialized host restriction reported to date for ISFs. Understanding this unprecedented host restriction in ISFs could help identify the mechanisms involved in the evolution of flaviviruses and their emergence as mosquito-borne pathogens.

## Abstract (precis)

Flaviviruses like dengue, Zika, or West Nile virus infect millions of people each year and are transmitted to humans via infected-mosquito bites. A subset of flaviviruses can only replicate in the mosquito host, and recent studies have shown that some can interfere with pathogenic flaviviruses in mosquitoes and limit the replication and transmission of the latter. The insect-specific flaviviruses (ISFs) reported here form a new *Anopheles* mosquito-associated clade separate from the *Aedes*- and *Culex*-associated ISF clades. The identification of distinct clades for each mosquito genus provides new insights into the evolution and ecology of flaviviruses. One of these viruses was shown to replicate in the midgut of the mosquito host and exhibit the most specialized host restriction reported to date for ISFs. Understanding this unprecedented host restriction in ISFs could help identify the mechanisms involved in the evolution of flaviviruses and their emergence as mosquito-borne pathogens.

## INTRODUCTION

The genus *Flavivirus* (family *Flaviviridae*) comprises enveloped, positive-sense single-stranded RNA viruses with a genome of approximately 10 to 11 kb contained in an icosahedral nucleocapsid (1–3). The viral RNA has 5′ and 3′ untranslated regions (UTRs) flanking a single open reading frame (ORF) coding for a single polyprotein composed of three structural proteins (capsid, membrane precursor/membrane, and envelope) and seven nonstructural proteins (NS1, NS2A, NS2B, NS3, NS4A, NS4B, and NS5) (4).

Most flaviviruses are arthropod borne (arboviruses) and cycle between an arthropod vector and a vertebrate host. They include medically significant viruses such as the Zika and dengue viruses. These viruses are usually transmitted horizontally during the feeding of a mosquito vector on a vertebrate host (5, 6). However, some flaviviruses have no known vector and have been isolated only from vertebrates, while others are termed insect-specific flaviviruses (ISFs), as they have been isolated only from mosquitoes, are unable to replicate in vertebrate cells, and cannot be transmitted via classical horizontal transmission (7). All evidence to date indicates that they are maintained in nature through vertical transmission (8–10).

ISFs form two distinct genetic lineages within the *Flavivirus* genus; classical ISFs (cISFs) cluster separately from vertebrate-infecting flaviviruses (VIFs) and include most of the species identified to date. Another group, divergent ISFs or dual-host-affiliated ISFs (dISFs), clusters more closely with VIFs (7). So far, cISFs have been isolated predominantly from *Aedes* or *Culex* mosquitoes and separate into two phylogenetic clusters according to the genus of their host. Only limited sequencing data have been reported for *Anopheles*-associated ISFs—one virus from Liberia with two full genome sequences (*Anopheles* flavivirus [AnFV]) and four partial sequences from Senegal and Kenya (*Anopheles squamosus* flavivirus [AnsFV] and *Anopheles gambiae* flavivirus [AngFV])—and none have been isolated or characterized (11, 12).

While all ISFs fail to replicate in vertebrate cells, *in vitro* and *in vivo* growth experiments have revealed variable host restriction for different mosquito genera. Indeed, Palm Creek virus (PCV), originally isolated from *Coquillettidia xanthogaster*, replicates effectively in *Culex* and *Aedes* species *in vitro* and *in vivo* (13, 14), while Parramatta River virus (PaRV), detected exclusively in *A. vigilax* mosquitoes, replicates only in cells of *Aedes* origin (15). This suggests that some ISFs have evolved to replicate efficiently in their mosquito host species but have either lost or never gained the ability to infect more distantly related species.

Most arbovirus families are thought to have evolved from insect-only to vertebrate-infecting life cycles (16–18). Similarly, recent evolutionary studies of flaviviruses suggest that cISFs constitute the ancestral forms from which the VIFs have evolved (19). To strengthen this theory, a broader collection of ISFs is required to allow a more comprehensive analysis. In addition to clarifying the evolutionary origins of the *Flavivirus* genus, such studies may also identify viral genetic elements associated with the transition from an arthropod to a vertebrate host.

Here we report the discovery of highly divergent flaviviruses specific to *Anopheles* hosts, likely forming the most ancient clade of ISFs detected to date. Our thorough characterization of one of these viruses, found in very high prevalence in *Anopheles meraukensis*, demonstrates that its replication is tightly restricted to this species.

## RESULTS

### Discovery of novel ISFs at high prevalence in *Anopheles* mosquitoes*.*

**FIG 1:**
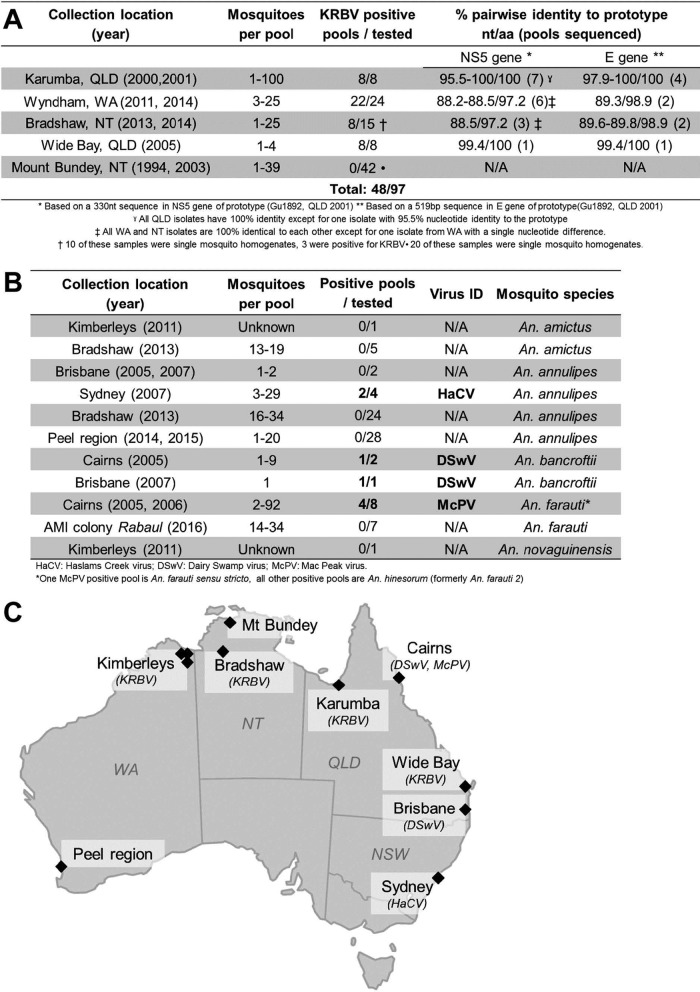
Detection of ISFs from *Anopheles* mosquitoes. Summary of *A. meraukensis* (A) and other *Anopheles* (B) pools tested. (C) Map of Australia showing the locations of mosquito trapping sites and the viruses detected.

As part of a study of insect-specific virus biodiversity in Australian mosquitoes, archival and recent mosquito homogenates were screened for ISFs. A 640-bp amplicon was produced by reverse transcription-PCR (RT-PCR) with a pair of pan-flavivirus primers (Flav100F/Flav200R) and RNA extracted from an *A. meraukensis* pool collected from the town of Karumba, Queensland, Australia (Fig. 1). Sequence analysis of this product identified a new ISF-like sequence from which specific primers were designed. Of 97 pools of *A. meraukensis* from various regions of Australia, 48 (49.5%) were positive for the virus sequence (Fig. 1), tentatively named Karumba virus (KRBV), with KRBV-specific primers and RNA extracted directly from the homogenate or from supernatant of inoculated C6/36 cells. When we assessed *A. meraukensis* mosquitoes collected from Wide Bay and Karumba in Queensland, the KRBV sequence prevalence was 100% (*n* = 16 pools), with some of the pools containing a single mosquito. In comparison, the prevalence of the KRBV sequences in mosquito pools from Western Australia (Wyndham) was 91.7% (*n* = 24) but it was substantially lower in mosquito populations of the Northern Territory, with detection in 53% of the mosquitoes collected in Bradshaw and no detection in *A. meraukensis* from Mount Bundey. It should be noted that 20 of the mosquito pools from Mount Bundey contained only a single mosquito (Fig. 1A).

All of the other *Anopheles* species tested (Fig. 1B) were negative for KRBV. The initial amplification of a KRBV sequence with pan-flavivirus primers could not be repeated with this primer pair (Flav100F/Flav200R) or a range of additional pan-flavivirus primers binding to the NS5 region or the 3′ UTR, i.e., FU2/cFD3, PF1S/2Rbis, and EMF1/VD8 (Table 1) (20–22), suggesting that KRBV is a very divergent flavivirus.

**TABLE 1:**
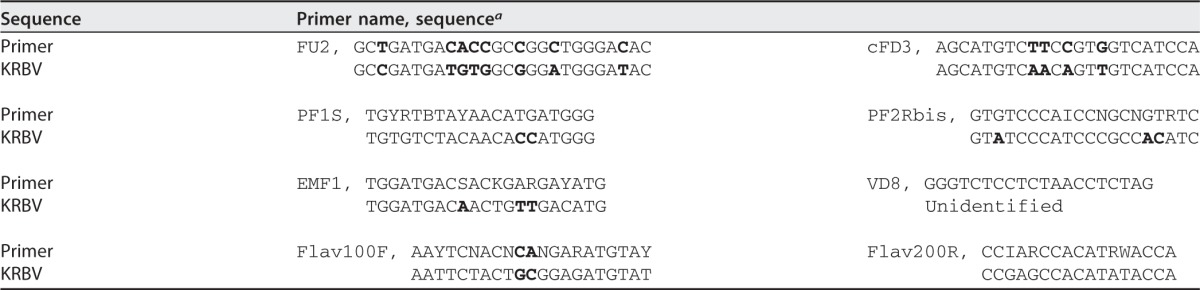
KRBV sequences at pan-flavivirus primer binding sites

a Boldface letters indicate differences between the primer and corresponding KRBV sequences.

Following the discovery of KRBV in *A. meraukensis* mosquito pools, other *Anopheles* mosquito species were tested for the presence of ISF RNA by RT-PCR with a newly designed cISF group-reactive primer pair (ISF F1/ISF R1). This resulted in the discovery of three additional novel partial NS5 sequences from *Anopheles annulipes*, *Anopheles bancrofti*, and *Anopheles farauti* mosquito pools. These viruses, tentatively named Haslams Creek virus (HaCV), Dairy Swamp virus (DSwV), and Mac Peak virus (McPV), respectively, were distinct from one another and most closely related to KRBV and other *Anopheles* ISFs.

### NGS of *Anopheles* mosquitoes reveals the presence of four new flavivirus species.

The entire genome sequence of KRBV was obtained by Illumina next-generation sequencing (NGS) for prototype sample 1892 (GenBank accession number KY460522). The KRBV ORF encodes a single polyprotein that consists of 3,342 amino acids (aa). The KRBV UTR lengths were typical for a flavivirus (5′ UTR, 115 nucleotides [nt] determined by NGS, of which 75 were confirmed by RT-PCR and Sanger sequencing; 3′ UTR, 505 nt determined by NGS, of which 500 were confirmed by RT-PCR and Sanger sequencing). Sequence alignments were performed over the ORF, revealing that the KRBV genome was most closely related to the only other published *Anopheles* ISF full-length genome, that of *Anopheles* flavivirus (AnFV), yielding 72.1% nucleotide sequence identity and 80.7% amino acid sequence identity. The closest non-*Anopheles*-associated relative of KRBV is Quang Binh virus, with 47.0% nucleotide sequence identity and 38.6% amino acid sequence identity.

Subsequently, full ORF sequences for McPV, HaCV, and a second strain of KRBV (Kim1, from the Kimberleys region of Western Australia) were obtained via Illumina NGS, as well as 5 kb of sequence forming the second half of the DSwV genome (GenBank accession number MF352618). Kim1 has 87.8% nucleotide sequence identity with the prototype strain of KRBV (isolate 1892) over the whole ORF nucleotide sequence and 95.4% similarity over the amino acid ORF sequence, indicating that it is a divergent strain of KRBV (Fig. 1A). The nucleotide and amino acid sequence identities of the new virus sequences with reference flaviviruses are summarized in Table 2 and clearly demonstrate that the four viruses found are distinct and novel flavivirus species.

**TABLE 2:** Nucleotide and amino acid sequence identities of novel *Anopheles* ISFs with reference flaviviruses^a^

Virus	% Identity with:																			
	1892	WBay2	Kim1	McPV	HaCV	DSwV^b^	AnFV	PaRV	CFAV	CxFV	QBV	PCV	BgV	WNV	ZIKV	DENV-2	LAMV	CHAOV	NOUV	BJV
1892		99.5	95.4	82.1	81.8	78.8	80.7	35.7	36.0	37.7	38.6	38.0	24.1	25.0	24.9	24.0	24.0	24.2	23.9	24.7
WBay2	99.4		95.5	82.1	81.8	78.9	80.7	35.7	36.1	37.7	38.6	38.0	24.2	24.9	24.9	23.9	24.0	24.2	23.9	24.7
Kim1	87.8	87.9		82.1	82.1	78.9	80.9	35.9	35.9	37.7	38.5	37.9	24.4	24.9	24.8	24.0	24.0	24.2	23.8	24.6
McPV	73.3	73.2	73.4		81.0	79.3	80.2	35.7	36.0	37.6	37.9	37.5	24.1	24.9	24.7	24.0	24.0	23.9	23.3	23.7
HaCV	72.6	72.6	73.1	73.7		79.6	79.2	33.4	33.3	36.0	36.2	35.5	23.2	23.9	23.8	22.9	22.9	22.8	22.5	23.3
DSwV^a^	69.7	69.8	69.2	71.8	72.9		77.7	39.7	40.7	43.5	43.4	43.4	29.9	29.8	30.0	30.4	29.4	29.3	29.8	29.9
AnFV	72.1	72.1	72.9	72.7	72.2	70.8		35.1	35.6	37.2	37.8	37.5	23.9	24.9	25.0	24.4	23.8	23.8	23.5	24.4
PaRV	44.8	44.9	44.9	44.3	43.7	47.0	44.7		41.8	38.7	38.0	38.3	25.1	24.8	24.7	24.4	24.4	24.5	24.5	24.9
CFAV	44.8	44.8	44.5	44.5	43.5	48.3	45.0	45.1		43.4	44.0	40.6	24.3	24.3	24.0	23.5	24.0	24.0	23.6	24.0
CxFV	46.8	46.8	46.3	46.3	45.4	50.1	46.5	45.0	48.4		64.4	51.9	23.7	24.3	24.4	23.9	23.2	23.2	22.8	23.7
QBV	47.0	46.9	46.8	46.8	44.8	49.2	46.5	44.4	48.7	61.9		53.1	23.9	23.9	24,4	23.7	23.5	23.8	23.5	23.9
PCV	47.0	47.0	46.6	46.6	45.3	49.6	46.8	44.1	46.3	52.4	52.8		23.9	24.2	23.9	23.8	23.8	23.9	23.0	23.5
BgV	37.3	37.3	37.4	36.9	36.5	39.6	37.1	35.8	35.2	35.1	35.6	35.9		44.3	45.2	43.3	44.8	44.9	43.2	44.5
WNV	37.0	37.0	37.3	37.1	36.2	39.9	36.9	36.0	36.1	35.9	35.8	36.0	49.1		56.9	51.6	49.5	49.8	46.9	49.2
ZIKV	38.1	38.1	37.9	37.7	37.4	41.0	38.3	35.9	36.0	36.0	35.6	36.1	48.2	55.3		54.9	49.8	49.7	48.1	49.6
DENV-2	37.6	37.6	37.9	37.0	36.6	40.3	37.3	35.5	35.4	35.0	35.2	34.7	48.8	52.1	55.4		46.7	46.8	44.5	46.8
LAMV	36.7	36.7	36.4	36.8	35.4	39.8	36.5	35.9	35.4	35.2	35.1	35.6	48.9	51.8	51.8	50.2		85.5	46.3	48.3
CHAOV	37.4	37.4	37.3	37.2	36.3	40.2	37.0	35.7	35.6	35.3	35.1	35.9	48.9	51.8	51.6	49.8	71.8		46.6	48.3
NOUV	36.9	36.9	36.8	36.9	36.1	40.4	37.1	35.5	35.4	35.5	35.1	35.7	47.9	50.0	51.5	49.4	49.9	50.1		53.2
BJV	37.8	37.9	38.0	38.0	37.2	39.7	38.0	36.1	36.4	36.1	35.8	36.0	48.4	51.2	50.5	49.3	50.2	50.2	53.7	

a The top right half is amino acid sequence identity, and bottom left half is nucleotide sequence identity. 1892, KRBV prototype strain; WBay2, KRBV strain; Kim1, KRBV strain; CFAV, cell fusing agent virus; CxFV, *Culex* flavivirus; QBV, Quang Binh virus; ZIKV, Zika virus; DENV-2, dengue virus serotype 2; CHAOV, Chaoyang virus; NOUV, Nounane virus.

b All sequence similarities are for the ORF, except for DSwV, as only the last 5 kb of the genome were available.

**FIG 2:**
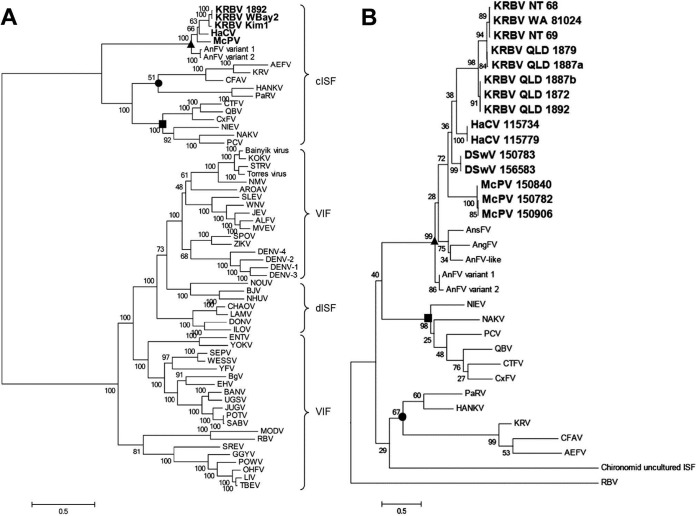
Phylogeny of novel ISFs. ML phylogenetic analysis of ORF amino acid sequence (A) and partial NS5 nucleotide sequence (~350 nt) of *Anopheles* ISFs aligned with other flaviviruses (B). The node highlighted with a disc comprises *Aedes*-associated ISFs. The node highlighted with a square comprises *Culex*-associated ISFs. The node highlighted with a triangle comprises *Anopheles*-associated ISFs.

The maximum-likelihood (ML) phylogeny of the ORF revealed that *Anopheles* ISFs cluster with cISFs and branch at a basal position within the clade, indicating the ancestral position of the common ancestor (Fig. 2A). KRBV also appears to form a distinct lineage with AnFV, HaCV, and McPV, separate from *Culex*- and *Aedes*-associated cISFs. The partial ISF nucleotide sequences obtained for all HaCV (GenBank accession no. KY460534 and KY460535), DSwV (KY460536 and KY460537), and McPV (KY460531 to KY460533) virus sequences were included in a partial NS5 gene alignment (~350 nt) along with several KRBV sequences from various locations (KY460524 to KY460530), and an ML tree further showed that the *Anopheles* ISFs cluster together, forming a single, separate clade (Fig. 2B).

Putative cleavage sites in the predicted KRBV, McPV, and HaCV polyprotein sequences revealed that their ORFs code for viral proteins according to the standard flavivirus genome organization (Table 3). The pr-M furin cleavage site for KRBV, McPV, and HaCV occurs between amino acids 172 and 173 and follows the consensus established for other flaviviruses (23), suggesting that the cleavage is efficient, unlike for some cISFs (e.g., PaRV) that exhibit inefficient pr-M cleavage (15).

**TABLE 3:** Predicted cleavage sites in the polyproteins of KRBV, McPV, HaCV, and DSwV

Protein cleavage^a^	Sequence^b^			
	KRBV	McPV	HaCV	DSwV
C/AnchC	TRQR ↓ TGNN	ARQR ↓ TGGN	TRQR ↓ TGGN	Unknown
AnchC/pr-M	LACA ↓ KTMN	FGCA ↓ KTMN	YGCA ↓ KTMT	Unknown
pr-M/M	RVKR ↓ GEPG	RVKR ↓ DQEG	RVKR ↓ DSAE	Unknown
M/E	VVQA ↓ SLAD	VVKA ↓ SLAD	IVQA ↓ SLAD	Unknown
E/NS1	YVRA ↓ DVGC	YVRA ↓ DVGC	YVRA ↓ DVGC	Unknown
NS1/NS2A	ESVA ↓ QPVT	ESNA ↓ QAVE	ESEV ↓ KPIT	Unknown
NS2A/NS2B	NWRR ↓ APAP	NWRR ↓ VPVP	NWRK ↓ VPVP	Unknown
NS2B/NS3	SCFR ↓ SDDG	SCFR ↓ SDDD	SCFR ↓ SDDG	Unknown
NS3/NS4A	LRMR ↓ AHIN	LRMR ↓ TSIN	LRMR ↓ ASVN	LRMR ↓ ASVN
NS4A/2K	SATR ↓ SYVD	SSTR ↓ SYVD	STTR ↓ SYVD	SSTR ↓ SYVD
2K/NS4B	GLVA ↓ FELD	GLVA ↓ FELD	GIVA ↓ FELD	GIVA ↓ FELD
NS4B/NS5	NSYR ↓ SSNK	SSTK ↓ GDAL	SSNK ↓ GDAL	SSNK ↓ GDAL

a C, capsid; AnchC, capsid anchor; pr-M, premembrane; M, membrane; E, envelope; NS, nonstructural protein; 2K, 2K peptide.

b Cleavage sites are indicated by downward arrows.

In addition to the main ORF coding for the three structural and seven nonstructural proteins, sequence analysis uncovered the presence of a predicted 879-bp coding sequence in the −1/+2 frame for KRBV, McPV, and HaCV, known to be present in ISFs as the fairly interesting flavivirus ORF (*fifo*) (24). The sequence starts with the conserved “slippery” RNA motif GGAUUUU for all three viruses, 53 nt downstream of the predicted cleavage between NS1 and NS2A. The motif is followed by six spacing nucleotides directly followed by a 34-bp stable RNA stem-loop (20 paired nucleotides for KRBV, 22 paired nucleotides for McPV, and 22 paired nucleotides for HaCV). These features are thought to stimulate a ribosomal frameshift that results in the translation of a 293-aa-long protein. *fifo* seems to be specific to cISFs and is present in all of the cISF sequences investigated to date. With two predicted transmembrane domains, McPV, HaCV, and KRBV (Kim1 strain) follow the consensus established for other ISFs, while KRBV (prototype strain) *fifo* appears to have only one transmembrane domain (11, 24).

**FIG 3:**
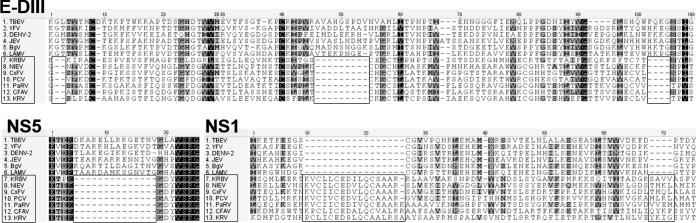
Alignment of KRBV and other flavivirus amino acid sequences displaying conserved deletions and insertions in the structural and nonstructural genes.

The cISFs genomes differ from VIF genomes by conserved insertions and deletions that may potentially be associated with ISF host restriction or enhanced vertical transmission (15). Similar conserved deletions and insertions were observed in both structural and nonstructural KRBV proteins (Fig. 3), supporting the affiliation of KRBV with the cISFs. McPV, HaCV, and DSwV also had these conserved deletions and insertions (data not shown).

### Nucleotide composition analysis confirms that *Anopheles*-associated ISFs have evolved in an insect host.

**FIG 4:**
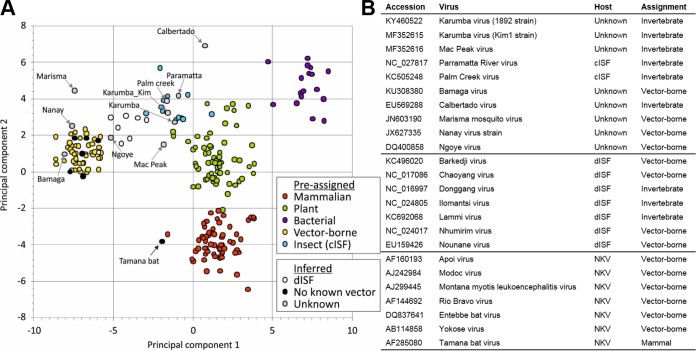
Nucleotide composition analysis. (A) Canonical score plot for the first two most influential components of discriminant analysis showing separation of groups according to host assignment. Points represent values for individual sequences. (B) Assignment of viruses with no defined host range by discriminant analysis.

Nucleotide and codon bias usage analysis has previously shown that flaviviruses have coevolved with their hosts: VIFs share the pronounced CpG underrepresentation observed in vertebrate genomes, while ISFs lack this frequency reduction, similar to the genomes of their insect hosts (25). These traits can be used to determine their host assignment. The frequencies of the 4 mononucleotides and 16 dinucleotides in virus sequences were analyzed by discriminant analysis, which predicted correct host assignments for all but one of the 197 control sequences and assigned hosts to viruses with poorly defined or unknown hosts (Fig. 4). Two strains of KRBV, HaCV, and other Australian cISFs, PCV and PaRV, grouped with the insect-only viruses. Assignments for dISFs varied between insect only and vector borne, with those phylogenetically related to Lammi virus (LAMV) being assigned to the insect-only group, and those related to Barkedji virus (BJV) assigned to the vector-borne group. Bamaga virus (BgV), another recently discovered Australian flavivirus with limited replication in vertebrates, grouped closely with VIFs, consistent with its phylogenetic position (23). It was noteworthy that the flaviviruses with no known vector generally grouped closely with vector-borne viruses, with the exception of the highly divergent Tamana bat virus sequence, which was predicted to be associated only with a vertebrate host in this analysis.

### *Anopheles* ISFs do not replicate in cells derived from heterologous species.

KRBV RNA was initially detected in the supernatant of C6/36 cells that had been inoculated with mosquito homogenate and incubated for 5 to 7 days at 28°C without removing the inoculum. This suggested that either the amplified sequence was from a virus replicating in the culture or the RNA was residual from the inoculum and likely protected from degradation in cell culture by encapsidation in a particle. No viral RNA could be reliably detected in subsequent passage of these samples on C6/36 cells (Table 4), suggesting that the initial detection was likely due to virion-protected RNA in residual inoculum and that the virus was unable to infect and replicate in C6/36 cells. In comparison, other mosquito samples collected at the same time and stored in the same way yielded numerous virus isolates by the same protocol, including PaRV from Sydney and PCV from Karumba, confirming that the mosquito samples had been adequately stored to yield culturable infectious flavivirus (15).

**TABLE 4:** Replication of flaviviruses *in vitro* and *in vivo*

Cells	Replication of:					
	KRBV	HaCV	McPV	DSwV	WNV^a^	BgV
C6/36	−	−	−	−	+	+
MOS55	−	−	−	−	+	+
S2	−	−	−	−	+	+
ISE6	−	−	−	−	NT^b^	+
BSR	−	NT	NT	NT	+	NT
Vero	−	NT	NT	NT	+	NT
DF-1	−	NT	NT	NT	+	NT
Injected *A. farauti*	−	NT	NT	NT	NT	NT

a Kunjin strain.

b NT, not tested.

The lack of replication of *Anopheles* ISFs *in vitro* was confirmed by inoculating KRBV-, HaCV-, McPV-, and DSwV-positive mosquito homogenates onto monolayers of mosquito (C6/36, *Aedes albopictus*; MOS55, *Anopheles gambiae*), tick (ISE6, *Ixodes scapularis*), and fruit fly (S2, *Drosophila melanogaster*) cells and incubating them for 2 h; the inoculum was removed, and the cells were washed prior to further incubation. No replication of *Anopheles* ISFs was detected after 5 to 7 days by virus-specific or generic ISF RT-PCR or with anti-double-stranded RNA (dsRNA) monoclonal antibodies (MAbs) in a fixed-cell enzyme-linked immunosorbent assay (ELISA) (Table 4) (26). The same lack of KRBV replication was observed in hamster (BSR, *Mesocricetus auratus*), monkey (Veros, *Cercopithecus aethiops*), and bird (DF-1, *Gallus gallus*) cells by KRBV-specific RT-PCR and immunofluorescence assay (IFA) with anti-dsRNA MAbs and anti-KRBV NS1 serum (Table 4). In comparison, the reference flaviviruses (West Nile virus [WNV] and BgV) replicated in all of the cell lines tested (Table 4). These results confirmed that *Anopheles* ISFs could not infect and replicate in commonly used arthropod or vertebrate cell lines, including C6/36 cells, which have a deficient RNA interference (RNAi) response (27) and have been shown to support the replication of all of the other mosquito-borne flaviviruses tested to date by the same method of isolation.

To assess whether KRBV could replicate *in vivo*, RT-PCR-positive *A. meraukensis* homogenates were inoculated intrathoracically into colonized *A. farauti sensu stricto* mosquitoes shown to be free of ISF RNA and tested for KRBV RNA at 3 h or 5 days postinjection in pools of three. KRBV sequence could be detected in 4/6 pools sampled within 3 h of inoculation, confirming the presence of detectable KRBV in the inoculum. However, no viral sequence was detected in six pools harvested 5 days postinjection, suggesting a lack of KRBV replication (Table 4).

### KRBV sequence was derived from an RNA template, not from integration into mosquito DNA.

**FIG 5:**
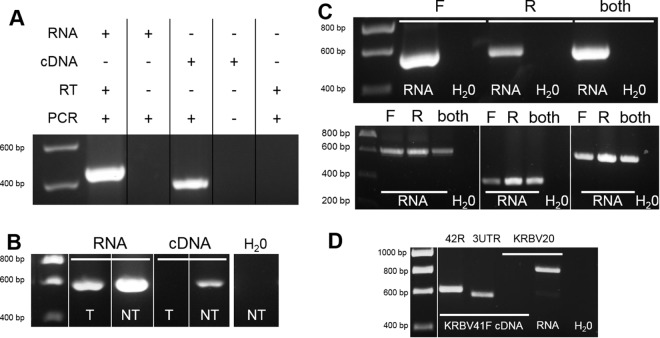
Detection of positive and negative KRBV genomic RNA strands as an indication of viral replication. (A) Amplification of KRBV sequence with and without RT with KRBV-specific primers. The first lane has KRBV RNA subjected to RT-PCR, the second lane has KRBV RNA subjected to PCR only, the third lane has KRBV cDNA subjected to PCR, the fourth lane shows the unamplified input cDNA, and the last lane shows the nontemplate control subjected to RT-PCR. (B) RT-PCR of DNase-treated KRBV RNA. T, DNase treated; NT, not treated. (C) RT-PCR amplification of *Anopheles* ISF sequences with forward only (F), reverse only (R), or both virus-specific primers during RT. Top gel, KRBV; bottom gel, from left to right, DSwV, McPV, and HaCV. (D) PCRs of cDNA generated with forward primer KRBV41F and with downstream (KRBV42R and KRBV3UTR1F/1R pair) and upstream (KRBV20F/R pair) primers and control RT-PCR of KRBV RNA for upstream primers.

To confirm that the KRBV sequences amplified by RT-PCR indeed originated from RNA, we performed simultaneous RT-PCR and PCR with KRBV-specific primers on the same RNA extract, as the RNA isolation kit used allows for DNA to be eluted along with purified RNA. KRBV sequence was amplified from mosquito homogenates only when an RT step was performed before the PCR, indicating that the amplification was from an RNA template (Fig. 5A). We also found that the KRBV template was still amplified by RT-PCR after RQ1 DNase digestion of KRBV RNA, confirming these findings (Fig. 5B).

Some ISF sequences have been shown to be integrated into their mosquito host genomes as the virus and host coevolve and are identified as endogenous viral elements (EVEs) (28–30). To assess whether the detected KRBV RNA template for amplification was similarly derived from transcripts of viral genes integrated into the genome of *A. meraukensis*, mosquito genomic DNA (gDNA) was tested for KRBV sequence by PCR with various combinations of virus-specific primers. Despite successful amplification of the conserved internal transcribed spacer 2 (ITS2) region in the repeated ribosomal DNA genes of the *Anopheles punctulatus* complex from gDNA of both *A. farauti* controls and *A. meraukensis*, no KRBV sequence was amplified with any of the KRBV-specific primer combinations used (data not shown). This implies that the KRBV sequence was amplified from a viral genomic RNA template in the mosquito and not from mRNA transcripts of viral genes integrated into host chromosomes. In addition, several *A. meraukensis* homogenates were found to be negative for the KRBV sequence by RT-PCR, suggesting that the viral sequence was unlikely to be integrated into the genome of this mosquito species.

### Detection of KRBV RNA replicative intermediates and proteins.

**FIG 6:**
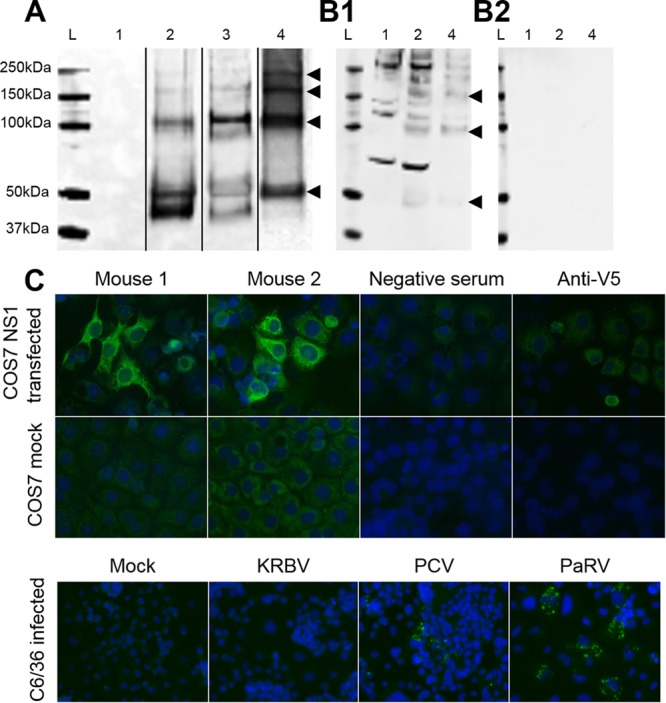
Production of recombinant KRBV NS1 and antiserum reactivity. SDS-PAGE and Western blot analysis of unboiled and unreduced untransfected COS-7L cell lysate (lanes 1); KRBV NS1-transfected COS-7L cell lysate (lanes 2), and concentrated supernatant (lanes 3); HisTrap column-purified KRBV NS1 (lanes 4) show NS1 monomers at approximately 50 kDa and oligomers at 100 and 150 kDa and higher molecular masses. Lanes L contain the molecular weight marker Precision Plus Protein Kaleidoscope (Bio-Rad). Panel A, with anti-V5 epitope MAb (lanes rearranged for figure clarity); panel B1, with immunized mouse serum (mouse 2); panel B2, with negative mouse serum. (C) IFA with anti-KRBV NS1 mouse serum of COS-7L cells (mock transfected and transfected with KRBV NS1) and C6/36 cells infected with cISFs (mouse 2 antiserum).

**FIG 7:**
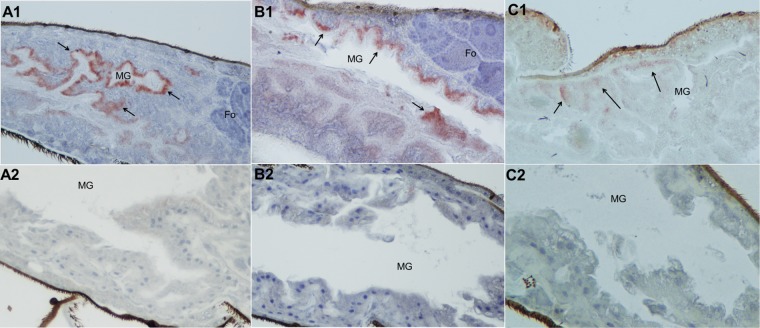
Detection of KRBV protein and replicative intermediates in *A. meraukensis* mosquitoes. Detection of KRBV NS1 (red signal) with MAbs 5G2 (A1) and 6F8 (B1) in midgut epithelial cells (MG). C1, faint signal for dsRNA with MAb 3G1 (arrows) in MG. Negative MG in mosquitoes on same slide as the KRBV-positive specimens labeled with 5G2 (A2), 6F8 (B2), and 3G1 (C2). Follicles (Fo) are negative for viral NS1 protein. It is important to note that NS1 accumulates in flavivirus-infected cells and is thus more abundant than the replicative intermediates. In addition, MAb 3G1 is an IgM, which can further explain the faint signal obtained.

To detect viral proteins in sections of naturally infected mosquitoes, we produced recombinant KRBV NS1 (rNS1) and generated mouse antiserum and MAbs to this protein. rNS1 displayed dimers and oligomers typical of a flavivirus NS1 protein by Western blotting (Fig. 6A), and its identity was confirmed by mass spectrometry (five peptides identified; data not shown). rNS1 elicited an antibody response in mice sufficient for its detection by Western blotting and in IFA (Fig. 6). The antiserum displayed cross-reactivity with other ISF NS1 proteins, reacting to both *Aedes*- and *Culex*-associated Australian ISF proteins (PaRV and PCV) in an IFA, supporting the classification of KRBV as a cISF (Fig. 6). MAbs derived from the immunized mice detected rNS1 in an ELISA and recognized the native viral protein in sections of 3 out of 10 *A. meraukensis* mosquitoes by immunohistochemistry (IHC) analysis, thereby showing expression of KRBV NS1 in these mosquitoes (Fig. 7). KRBV-NS1 expression was restricted to epithelial cells of the midgut in the absence of overt cytopathology, and the protein colocalized with viral dsRNA detected with anti-dsRNA MAbs, suggesting KRBV replication in the mosquitoes (Fig. 7).

To provide further evidence of the presence of viral dsRNA and therefore replication of KRBV in *A. meraukensis* tissues, KRBV sequence was amplified from cDNA transcribed from both positive and negative RNA strands (Fig. 5C). A similar experiment was performed with McPV-, HaCV-, and DSwV-positive homogenate RNA and virus-specific primers, and it provided evidence of the presence of dsRNA replicative intermediates in these *Anopheles* homogenates as well (Fig. 5C; Table 5). To confirm that the PCR product was generated from cDNA transcribed from the negative strand and to eliminate the possibility that the cDNA was generated from mispriming of KRBV41F on the positive strand, downstream of the KRBV41F/42R binding site, we designed primers upstream of the KRBV41F/42R site (KRBV20F/R) and downstream, at the very end of the KRBV genome (KRBV3UTR1F/KRBV3UTR1R). We showed that the upstream primers failed to amplify a product while the downstream primers successfully amplified a PCR product from the cDNA generated with KRBV41F from the negative strand (Fig. 5D).

**TABLE 5:** *Anopheles* ISF primers used in this study

Primer^a^	Sequence	Used^b^ with:
KRBV2F	ACATTGCCGACAGGGACACG	2R
KRBV2R	CCAACAGCTGCATCTGAACG	2F
KRBV41F	GGTCTTGTTTGCGCCTTCATGTGC	42
KRBV42R	CGCGTTTGTTATTCTTGGCTTCC	41
KRBV7F	CCAAACTCGTACCGGTCATCAAAC	7R, 8R
KRBV7R	GCCATAAGTCATGAACGCCTCG	7F, 8F
KRBV8F	CGGAATATCAACCAGGGGATTGTG	8R, 7R, 9R
KRBV8R	GCTAACCAATTTTCCAACAGGGTG	8F, 7F, 9F
KRBV9F	CCAATTAACCTTCGTCACAGCTGC	9R, 8R, 10R
KRBV9R	GCAGTGGAATTTCTACTTAAGCGC	9F, 8F, 10F
KRBV10F	CGGATCGACCATCCCTAGAAAGAG	10R, 9R
KRBV10R	CGTTAGAGCTCTCAATGTTGC	10F, 9F
KRBV20F	TCATGGAGCATATGCATTCG	NA^c^
KRBV20R	TCAGTGACTTCAGATCCTCC	NA
KRBV3UTR1F	CGACGTGTCTTGGACAAACACG	NA
KRBV3UTR1R	CCTGCCTGTGTTTTCTTGG	NA
DSwV1F	CGACGTGAATATGGCAAAGG	NA
DSwV1R	CACACTAGCTCTCATCCTGAGC	NA
McPV3502F	CCCTGACGTTGTATTGGTACC	NA
MCPV3875R	GCTAGCTGCGAGATATGTGC	NA
HaCV2833F	CACATGCCTGGGTATCACACG	NA
HaCV3393R	CGACACCAATAAGGACTGTCC	NA
ISF F1	GGGCAAGTARBMACTTATGCVTTGAACAC	NA
ISF R1	GCCCACATCTGGGCRTRNGCCTTNGC	NA

a F, forward; R, reverse.

b Combination(s) used for gDNA detection.

c NA, not applicable.

### Detection of *Anopheles* ISF-specific siRNA provides evidence of the mosquito immune response to viral infection.

**FIG 8:**
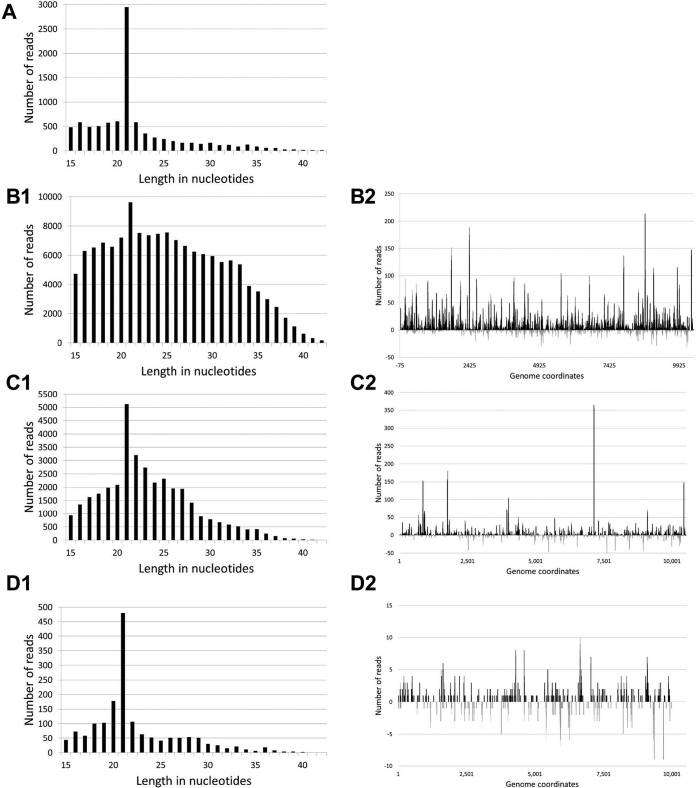
Analysis of siRNA in mosquito homogenates. Size distribution of mapped reads: PaRV-positive *A. vigilax* homogenate (A), KRBV-positive *A. meraukensis* homogenates (B1 and C1), and HaCV-positive *A. annulipes* homogenate (D1). Distribution of 21-nt reads over the genome sequences of KRBV-positive *A. meraukensis* homogenates (B2 and C2) and HaCV-positive *A. annulipes* homogenate (D2) with top reads mapping to the sense strand of the genome and bottom reads mapping to the antisense strand of the genome.

Small RNA (sRNA) reads generated by NGS of mosquito homogenates positive for KRBV, HaCV, or PaRV were aligned with the corresponding reference ORF to identify virus-specific siRNA (vsiRNA) produced in response to replicating virus. Reads that mapped to the virus genomes displayed a peak at 21 nt for all samples, which corresponds to the size of siRNAs generated via the cleavage of viral dsRNA by dicer-2 as part of the mosquito RNAi response to a replicating virus (Fig. 8A) (31, 32). The 21-nt reads mapped to both the sense and antisense strands of the KRBV and HaCV genomes, with a majority mapping to the sense strand, consistent with the expected response for replicating viruses (Fig. 8B to D). The assembly of vsiRNA reads produced an ORF sequence for a third KRBV strain (WBay2) in addition to the prototype strain and KRBV strain Kim1, and it was included in the phylogenetic analysis (Fig. 2A). WBay2 has 99.4% nucleotide sequence and 99.5% amino acid sequence identity with the prototype strain of KRBV (1892) over the whole ORF sequence.

## DISCUSSION

We report here the discovery of four novel ISFs exclusive to an *Anopheles* host. These viruses are highly prevalent in several *Anopheles* populations, with infection rates of up to 100% in mosquito pools from some cohorts and frequent detection in homogenates of single mosquitoes. In a cohort of 10 single mosquitoes collected from Bradshaw, 3 were shown to be positive for KRBV RNA. Similarly, KRBV NS1 protein was detected in 3 out of 10 mosquitoes by IHC analysis. We detected KRBV in *A. meraukensis* pools from multiple years in traps located as far as 2,800 km apart. This study is the first to demonstrate the presence of an *Anopheles* ISF with a widespread geographical and temporal distribution at a high prevalence. The findings suggest that the virus became established in *A. meraukensis* populations a long time ago and was maintained via a highly efficient mode of vertical transmission.

According to the criteria for species demarcation within the *Flavivirus* genus, as proposed in the 2016 report by the International Committee for the Taxonomy of Viruses (https://talk.ictvonline.org/ictv-reports/ictv_online_report/positive-sense-rna-viruses/w/flaviviridae/360/genus-flavivirus), which includes nucleotide and amino acid sequence identities, host and vector associations, and geographical distributions, KRBV, HaCV, McPV, and DSwV should be considered new species. These new virus sequences also group phylogenetically with other cISFs but form a separate *Anopheles*-associated clade distinct from *Culex*- and *Aedes-*associated cISFs. We propose a new designation for ISFs, with cISFs becoming lineage I (IA for *Aedes*-associated ISFs, IB for *Anopheles*-associated ISFs, and IC for *Culex*-associated ISFs) and dISFs becoming lineage II, as outlined by Hall et al*.* (33). The *Anopheles* clade (or lineage IB) appears to be more closely related to other lineage I ISFs than to other flaviviruses, including an ISF-like partial sequence discovered in chironomid species (34), demonstrating that the *Anopheles* viruses most likely replicate in mosquitoes, rather than being derived from host microbiota or parasites. In addition, the recent discovery of EVEs in *Anopheles minimus* and *A. sinensis* supports our conclusion that *Anopheles* mosquitoes are natural hosts of ISFs (30). Since most EVEs appear to be integrated into the host genome early during the host-virus evolutionary relationship, it is likely that some KRBV sequences have been integrated into the *A. meraukensis* genome at some time in the past, despite our inability to detect them (30). Sequencing of the *A. meraukensis* genome could shed light on the presence of ISF EVEs in this mosquito genome.

Nucleotide motif usage analysis has previously shown that flaviviruses have coevolved with their hosts: VIFs share the pronounced CpG underrepresentation observed for vertebrate genomes, while ISFs lack this frequency reduction, similar to their insect hosts’ genomes (25). This reflects the avoidance of CpG in vertebrates to reduce the chance of C-to-T mutations due to spontaneous deamination of cytosine by methylation at these sites, a process that does not occur in insects (35). Like other ISFs, KRBV and McPV sequences display an insect-like nucleotide usage bias. The fact that ISFs are not constrained to mimic the dinucleotide bias of vertebrates provides further evidence of their adaption to a mosquito-only transmission cycle.

Separate clustering of viruses according to mosquito genera would suggest coevolution of the viruses and their hosts, at least to some extent. The basal phylogenetic position of the *Anopheles* ISF clade supports this theory, as *Anopheles* is considered the oldest mosquito genus, being the first to have diverged from other mosquito genera (36, 37). Coevolution of the *Anopheles* ISFs with their host species provides an explanation for the narrow host restriction of these viruses and is, to some degree, corroborated by our data, as each virus was detected in a single *Anopheles* species. This does not preclude the possibility of several viruses being found in the same species in other mosquito populations; two different ISF sequences were detected in *A. gambiae* from Kenya and Senegal, approximately 8,000 km apart (11, 12).

Although the new *Anopheles* ISFs group phylogenetically with other lineage I ISFs, they seem to be genetically and phylogenetically divergent from other flaviviruses. None of these viral sequences were amplified by generic flavivirus primers, which collectively recognize all of the other members of the genus tested to date, including other ISFs. The *Anopheles* ISFs could be detected only by a set of generic primers based on lineage I ISF sequences. KRBV has only ~40% sequence identity with its closest non-*Anopheles*-associated relative, Quang Binh virus, a lineage IC ISF from Vietnam. Furthermore, although we showed that KRBV replicates in its natural host, we were unable to find a laboratory model to support KRBV replication, including *A. albopictus* and *A. gambiae* cell lines, despite the former being particularly permissive to infection with flaviviruses and the latter being derived from the same genus as the natural host of the viruses. Viruses from various families, including flaviviruses, were isolated from the same cohorts of samples as part of separate studies, showing that the homogenates were appropriately stored and processed for virus isolation (15, 33, 38). Moreover, attempts to infect colonized *A. farauti sensu stricto* mosquitoes with KRBV were unsuccessful. These findings contrast with other studies performed with ISFs. PCV and CxFV replicated efficiently after intrathoracic inoculation into mosquitoes of different species and genera (13, 39). A key experimental difference was the use of KRBV-positive homogenates as an inoculum in this study, since a pure virus stock could not be generated. The viral dose may have been suboptimal, or nonviral constituents of the homogenate may have stimulated an innate immune response, abrogating replication of the virus. We were not able to assess the replication of KRBV in *A. meraukensis* mosquitoes, as we did not have access to a laboratory colony of this species, and acquisition of wild-caught *A. meraukensis* from its natural habitats in remote regions of Australia was not logistically possible. In any case, on the basis of our current data, wild-caught *A. meraukensis* would likely have a naturally high prevalence of KRBV infection. Nevertheless, together, our *in vitro* and *in vivo* findings suggest that *Anopheles* ISFs may be uniquely specific to their natural host species.

This unprecedented host restriction may be due to the virus’s reliance on host components absent from both cell culture and other mosquito species, originating either from the host itself or from a coinfecting parasite or virus, which would render *in vitro* and *in vivo* models unsuitable. One explanation may link host restriction with interactions during virus attachment and entry into the cell, with a potentially strong adaptation of the virus to a cell receptor specific to *A. meraukensis*.

Recent studies in other virus-insect host systems have demonstrated that while the viruses could suppress the RNAi defenses of their host species and allow efficient replication in the insect, they were unable to suppress the corresponding RNAi response in related host species and consequently failed to replicate (40). This evidence of species-dependent RNAi suppressors in insect viruses suggests that the species-specific tropism exhibited by KRBV may be due to its inability to sufficiently suppress the RNAi response in other *Anopheles* species. However, the presence of 21-nt-long siRNAs in *A. meraukensis* homogenates that map to the KRBV sequence shows that KRBV does not completely suppress the *A. meraukensis* RNAi response. In addition to this, no KRBV replication was detected, even in dicer-2-deficient C6/36 cells, which have an impaired siRNA response. Nevertheless, the RNAi response in insects comprises three pathways mediated by sRNAs, the siRNA, microRNA (miRNA), and Piwi-interacting RNA (piRNA) pathways (41). Consequently, while siRNA is thought to be the main cellular antiviral innate immune response in arthropods (42), the restricted tropism of KRBV may be explained by its inability to suppress other RNAi pathways, such as piRNA or miRNA pathways, in mosquito species other than its main host species. In any case, this unique host restriction limits the possibilities of virus characterization and is likely one reason why only sequence data for *Anopheles* ISFs have been published to date.

We attempted to detect KRBV proteins in mosquito homogenates by mass spectrometry but were unsuccessful, even in those from our control mosquitoes, *A. aegypti* injected with PaRV, suggesting that the limit of detection of the assay was too low to detect ISF proteins. However, we were able to detect KRBV NS1 in naturally infected mosquitoes by IHC analysis, indicative of viral replication, and this was corroborated by colocalization of anti-dsRNA antibodies, also by IHC analysis. In addition to proving natural infection of mosquitoes by KRBV, these data show that the virus appears to be confined to the epithelial cells of the mosquito midgut, a narrow host cell tropism shared with other ISFs (13), which suggests that this characteristic is not linked to the unique host restriction of KRBV. Moreover, this localization is consistent with a mode of transmission from mother to progeny that does not necessarily involve replication in mosquito reproductive organs. Rather, it could involve indirect transmission of the virus to the egg prior to egg deposition, as shown by Jia et al*.* for rice dwarf virus in *Nephotettix cincticeps* leafhoppers (43).

Despite our inability to culture the *Anopheles* ISFs in either *in vitro* or *in vivo* systems, we have shown that KRBV, McPV, and HaCV possess a complete viral genome with a functional ORF. Furthermore, according to the dinucleotide usage pattern of their genomes, KRBV and McPV have evolved to replicate only in insects. We have also provided compelling evidence that *Anopheles* ISFs replicate in their host mosquitoes by demonstrating the presence of double-stranded replicative RNA forms, vsiRNA generated by the mosquito RNAi response, and KRBV-specific protein in mosquito tissues, specifically, midgut epithelial cells. Finally, our inability to amplify KRBV sequences from the *A. meraukensis* genome and the existence of KRBV-negative samples of this species effectively show that the KRBV sequence detected were not derived from EVEs present in the mosquito host’s genome. The characterization of these new *Anopheles* flaviviruses necessitated the use of a range of novel techniques and research tools that will enhance our understanding of the ecology, biology, and evolution of this unique group of flaviviruses.

## MATERIALS AND METHODS

### Trapping and processing of mosquitoes.

All of the mosquito samples tested in this study were archival and recent mosquito pools of the *Anopheles* genus collected for various studies (University of Western Australia, Army Malaria Institute; details are shown in Fig. 1) (38, 44). All mosquito homogenates were stored at −80°C to ensure proper conservation of potential viruses and RNA, quickly thawed at 37°C, and subsequently kept on ice before cell inoculation (see below).

### Screening of homogenates for novel flaviviruses.

Two hundred microliters of mosquito homogenate was used to inoculate C6/36 cell monolayers, maintained as described below, in 4 wells of a 96-well plate. Cultures were incubated for 5 to 7 days at 28°C, supernatant was harvested, and RNA was extracted from this supernatant with the NucleoSpin Viral RNA isolation kit from Macherey-Nagel. The original detection of the partial KRBV genome was performed by RT-PCR (Superscript III One-Step RT-PCR System with Platinum *Taq* DNA polymerase; Invitrogen) with generic NS5 flavivirus primers Flav100F/Flav200R (45). Subsequent screening was performed by RT-PCR with generic ISF primers from an alignment of the flavivirus NS5-encoding gene, including ISF sequences (ISF F1/ISF R1; Table 5) or virus-specific primers based on the short KRBV sequence available. When samples yielded an RT-PCR product of the expected size, its sequence was obtained by Sanger sequencing at the Australian Genome Research Facility (AGRF; Brisbane, Queensland, Australia) after amplicons were purified by agarose gel electrophoresis and extracted with the NucleoSpin Gel and PCR cleanup kit (Macherey-Nagel). Species-specific primers were subsequently designed by hand or with Primer3 in Geneious 8.0.5.

The mosquito species of positive pools were confirmed by sequencing of PCR products obtained with ITS2A/ITS2B primers (46). The RNA isolation kit allows for elution of DNA along with RNA, so these extracts were used as templates that were amplified by PCR with the One-Step RT-PCR System (Invitrogen).

### Sequence analysis and phylogenetics.

The complete sequence of KRBV was obtained from the prototype isolate (1892) RNA by Illumina NGS at the Roslin Institute, University of Edinburgh (United Kingdom), on a HiSeq 2000 platform. The raw sequencing data were analyzed with Geneious 8.0.5 with default settings for mapping to a reference. Paired reads were first mapped to the short KRBV sequence available, and the consensus sequence of this assembly was used as a mapping reference for subsequent assembly. The partial KRBV sequence and other partial ISFs sequences were obtained and confirmed by Sanger sequencing at AGRF. Whole-genome sequences for McPV, HaCV, and KRBV (Kim1), as well as 5 kb of DSwV sequence, were obtained by Illumina NGS on a HiSeq platform at AGRF (Melbourne). The raw sequencing data were also analyzed with Geneious, and any gaps in the sequences were confirmed by using virus-specific primers in a Superscript III one-step RT-PCR with Platinum *Taq* DNA Polymerase High Fidelity (Thermo, Fisher Scientific) and Sanger sequencing at AGRF (Brisbane). Alignments were performed with MUSCLE. Phylogenetic trees were constructed by ML with a GTR substitution model for 33 partial NS5 nucleotide sequences and an LG+G substitution model for 62 ORF amino acid sequences, 100 bootstrap replicates, and midpoint rooting (47). Putative cleavage sites in the virus polyprotein were predicted in accordance with previously described guidelines (4, 48). The furin cleavage site was determined with ProP 1.0 (http://www.cbs.dtu.dk/services/ProP/), and transmembrane domains were determined with the transmembrane domain prediction tool in Geneious 8.0.5.

### Nucleotide composition analysis.

Complete genome sequences from a set of 197 RNA viruses of supergroup II (*Flaviviridae*, *Tombusviridae*, *Umbravirus*, *Leviviridae*) with a defined host range (vertebrate only, invertebrate only [cISF], vector borne, plant, and bacterial) were used for nucleotide composition analysis. The mononucleotide and dinucleotide frequencies of each sequence were determined with the Composition Scan program in SSE version 1.1 (49). Dinucleotide biases were determined as the ratio of the observed frequencies of each of the 16 dinucleotides to the expected frequencies determined by multiplying the frequencies of each of the two constituent mononucleotides. The frequencies of each mononucleotide and dinucleotide (20 parameters) were used as predictive factors to infer the host ranges of unknown virus sequences by discriminant analysis as implemented in the statistical package Systat with default parameters as previously described (35). The two mononucleotide or dinucleotide frequencies chosen as parameters to plot the result in Fig. 4A (*x* and *y* axes) were the two frequencies that influenced the result the most.

### Replication *in vitro.*

The cell lines used were C6/36 (*A. albopictus*, RPMI 1640 medium, 2 to 5% fetal bovine serum [FBS], 28°C), MOS55 (*A. gambiae*, Schneider’s *Drosophila* medium, 2 to 10% FBS, 28°C), ISE6 (*I. scapularis*, supplemented L-15, 10% FBS, 34°C), S2 (*D. melanogaster*, Schneider’s *Drosophila* medium, 5% FBS, 28°C), BSR (*M. auratus*, Dulbecco’s modified Eagle medium [DMEM], 2 to 5% FBS, 37°C), Vero (*C. aethiops*, DMEM, 2 to 5% FBS, 37°C), and DF-1 (*G. gallus*, DMEM, 2 to 10% FBS, 37°C) (50, 51). Cell media were supplemented with 50 U/ml penicillin, 50 µg/ml streptomycin, and 2 mM l-glutamine.

Monolayers were inoculated with RT-PCR-positive *Anopheles* homogenates and incubated for 2 h at 28 or 37°C. The inoculum was then removed, and the cells were washed three times with sterile phosphate-buffered saline (PBS) and topped up with fresh medium. After incubation for 5 to 7 days, cells were fixed in 20% acetone in PBS with 0.02% BSA or 100% ice-cold acetone, RNA was extracted from the cell culture supernatants as described above and tested by RT-PCR with ISF generic or virus-specific primers. The fixed monolayers were tested for viral replication by fixed-cell ELISA or IFA with anti-dsRNA MAbs (MAVRIC) or KRBV NS1 antiserum in accordance with a previously described protocol (26).

### Replication *in vivo.*

A total of 201 female *A. farauti sensu stricto* mosquitoes from a colony established at the Australian Army Malaria Institute (AAMI) with mosquitoes from Rabaul, Papua New Guinea, were inoculated with approximately 220 nl of one of nine KRBV RT-PCR-positive *A. meraukensis* homogenates with a Nanoject II (Drummond Scientific, Broomall, PA) microinjector. A subset of each group was collected on the day of inoculation. The remaining mosquitoes were maintained at 28°C in high humidity with a 12-h dark–12-h light cycle in an environmental growth cabinet (Sanyo Electric, Gunma, Japan) and provided 15% honey water as a nutrient source. Pools of three mosquitoes harvested at 5 days postinjection were homogenized in 500 µl of RPMI medium supplemented with 2% FBS with a glass bead in a Tissue Lyser III (Qiagen) for 3 min at 30 Hz. RNA was extracted and tested by RT-PCR with virus-specific primers in accordance with the procedures described earlier.

Absence of an ISF persistently infecting the colony was tested for by RT-PCR with the generic ISF primers and five homogenates of 21 to 25 pooled larvae and one homogenate each of 14 female and 34 male 1- to 2-day-old adults.

### Detection of viral RNA and replicative forms.

KRBV-positive RNA was used in simultaneous RT-PCR and PCR with virus-specific primers. All of the conditions and buffers used were the same for the PCR as for the RT-PCR, except that the template was added after the RT step of the cycle for the PCR sample. A DNA template was used as a positive control for the PCR under these conditions. This template was viewed alongside the RT-PCR and PCR products to confirm that the sequence was indeed amplified by the PCR.

KRBV RNA was treated with RQ1 DNase (Promega) in accordance with the manufacturer’s protocol, at 37°C for 1 h. The DNase was then inactivated with stop solution at 65°C for 10 min, and the RNA was used as the template in an RT-PCR with KRBV-specific primers.

Replicative intermediates were detected in KRBV, McPV, HaCV, and DSwV homogenate RNA by RT-PCR with either a forward or a reverse virus-specific primer for the RT step and addition of the second primer only after the RT step of the cycle. The primers used are detailed in Table 5. They were KRBV41F/KRBV42R (559 bp in NS4), DSwV1F/DSwV1R (640 bp in NS3), McPV3502F/MCPV3875R (373 bp in NS2), and HaCV2833F/HaCV3393R (561 bp in NS1/NS2).

We confirmed that KRBV41F was not mispriming on the positive strand by generating cDNA with this primer as described above and by using both upstream and downstream primers to amplify the template by PCR. The downstream primers used were KRBV42R, as described above, and KRBV3UTR1F/KRBV3UTR1R (534 bp in the 3′ UTR). The upstream primer pair used was KRBV20F/KRBV20R (748 bp in NS3), and it was confirmed to bind the KRBV template by RNA amplification by RT-PCR.

### Testing of mosquito gDNA.

gDNA was extracted from single *A. meraukensis* mosquito bodies by two methods, the Invitrogen PureLink gDNA kit (used in accordance with the manufacturer’s protocol) and a previously published method (52). Single *A. farauti* mosquitoes were similarly processed as controls. The gDNA extracts were used in PCRs with the ITS2 primers as quality control and subsequently tested for integrated virus sequence with a range of primers based on KRBV NS4 and NS5 sequences. Six primer sets were used in 12 combinations to prevent false-negative results due to potential introns in the primer binding regions (Table 5). PCRs were performed with *Taq* DNA polymerase and Thermopol buffer (New England Biolabs) in accordance with the manufacturer’s protocol.

### Expression of rNS1 in mammalian cells.

The NS1-encoding gene was amplified by high-fidelity RT-PCR from RNA extracted from KRBV prototype sample 1892 and cloned into a mammalian expression vector by infusion cloning (Clontech). The 3′ end of the gene was fused to the V5 epitope sequence followed by a polyhistidine sequence. Sequencing of the expression plasmid confirmed that the recombinant gene fragment was authentic and in frame for correct translation. COS-7L cells were transfected with Lipofectamine 3000 (Invitrogen) in accordance with the manufacturer’s protocol. After 3 days, the cells were harvested with a cell scraper into PBS with protease inhibitors. rNS1 was purified on a HisTrap column (GE), buffer exchanged into PBS, and concentrated on an Amicon Ultra centrifugal filter with a 10-kDa cutoff (Merck Millipore). The presence of rNS1 was confirmed by Western blotting of cell lysate, cell supernatant, and purified protein and IFA of transfected cells with an anti-V5 MAb (Invitrogen) as described in reference 14. The identity of KRBV NS1 was confirmed by mass spectrometry in accordance with previously published protocols (53).

### Production of polyclonal serum and MAbs against KRBV NS1 in mice.

Two mice were injected intradermally with two doses of expression plasmid (20 µl per earlobe at 892 ng/µl) 2 weeks apart. Four weeks after the second plasmid injection, the mice received a subcutaneous injection of rNS1 with ADVAX adjuvant (54). This was followed by an additional two rNS1-plus-adjuvant injections 3 to 4 weeks apart. Mouse blood was obtained from the tail vein, and seroconversion was tested by Western blot assays of transfected cell lysate and purified rNS1 and by IFA of transfected cells. When seroconversion was deemed sufficient, 7 weeks following the last boost, one mouse received a final intravenous injection of 50 µl of purified protein and was exsanguinated by cardiac puncture 4 days later. The spleen was harvested, and myeloma fusion was performed as described previously (55). All animal procedures were conducted in accordance with the Australian Code for the Care and Use of Animals for Scientific Purposes, 8th edition (2013), and were approved by the University of Queensland Institutional Animal Ethics Committee (AEC approval no. SCMB/329/15/ARC).

### IHC analysis.

Sixteen *A. meraukensis* mosquitoes collected from Bradshaw (AAMI) in 2014 and initially stored at −80°C were fixed in 4% formaldehyde–0.5% Triton X-100 in PBS for 24 h before their legs and wings were removed and their bodies were transferred to 70% ethanol for storage until routine processing to paraffin embedding. Only 10 of these 16 mosquitoes were in good enough condition to be stained after embedding and sectioning. Five-micrometer sections collected on charged slides were immunolabeled for dsRNA with MAVRIC and for KRBV NS1 with the specific mouse serum and MAbs 6F8 and 5G2 in accordance with a previously described protocol (13).

### siRNA detection.

siRNA was extracted from 150 to 210 µl of one PaRV-positive *A. vigilax* homogenate of 94 mosquitoes, one pool of KRBV-positive *A. meraukensis* homogenates from Wide Bay (WBay2, 2005) with 1 to 4 mosquitoes, one pool of KRBV-positive *A. meraukensis* homogenates from the Kimberleys (Kim1, 2014) with 25 mosquitoes, and an HaCV-positive homogenate of 29 *A. annulipes* mosquitoes from Sydney (2007) with the mirVana miRNA Isolation kit (Thermo Fisher Scientific) in accordance with the manufacturer’s protocol. One recovered fraction contained total RNA depleted of sRNA species, while the final fraction recovered was enriched in sRNA. The total RNA fraction was tested for PaRV, KRBV, and HaCV, respectively, by RT-PCR with virus-specific primers. sRNA samples were sequenced at AGRF by Illumina sequencing on a HiSeq platform.

CLC Genomics Workbench (version 7.0.4) was used to remove adapter sequences and reads with low quality scores from the data sets. We applied a quality score of 0.05 and a maximum of two ambiguous nucleotides as the cutoff for trimming. Reads without 3′ adapters and those <15 nt long after trimming were discarded. The clean sRNA reads were mapped to PaRV, KRBV, or HaCV sequences on the basis of strict mapping criteria (mismatch, insertion, and deletion costs of 2, 3, and 3, respectively). A minimum similarity and length fraction of 0.9 between a mapped segment and the references was allowed as part of the mapping criteria.

### Accession number(s).

The GenBank accession numbers for the viruses sequenced in this study and reported here are KY460522 to KY460537, MF352615, MF352616, MF352617, and MF352618.
